# Urinary Incontinence and Quality of Life in Women of Central Jordan: A Cross-Sectional Study

**DOI:** 10.3390/clinpract14050152

**Published:** 2024-09-20

**Authors:** Rana Abu-Huwaij, Rolla Al-Shalabi, Enas Alkhader, Farah N. Almasri

**Affiliations:** 1 College of Pharmacy, Amman Arab University, Amman 11953, Jordan; 2 Department of Toxicology, Advanced Medical and Dental Institute, Universiti Sains Malaysia, Gelugor 11700, Malaysia; rolaalshalabi@student.usm.my; 3 Faculty of Pharmacy, Middle East University, Amman 11831, Jordan; ealkhader@meu.edu.jo; 4 Faculty of Medicine, Jordan University of Science and Technology, Irbid 22110, Jordan; fnalmasri15@med.just.edu.jo

**Keywords:** women, quality of life, urinary incontinence, prevalence, Jordan

## Abstract

Background: Considering the high prevalence of UI in the rural areas of Jordan and the limited clinical data on its occurrence in central Jordan, this study aims to investigate the prevalence, risk factors, and impact of urinary incontinence (UI) on the quality of life (QoL) of women in central Jordan. Method: This cross-sectional study was conducted from September to December 2022, using online the Incontinence Impact Questionnaire short form (IIQ-7) and Urogenital Distress Inventory short form (UDI-6). Participation was voluntary, and anonymous. Internal consistency was assessed using Cronbach’s α. Results: A total of 128 women participated in the study. More than half of the participants (54.33%, N = 69) experienced UI symptoms. Body mass index was the sole statistically significant factor linked to UI. Obese patients had the highest risk (OR 35, CI 95% 2.577–475.308, *p* < 0.05) compared to those with a healthy weight. Multivariate regression indicated significant associations of severe UI with smoking and vaginal births with a moderate impact of UI on QoL. Conclusions: The study’s findings emphasize the need for women’s health centers in the center of Jordan to develop comprehensive UI prevention and management programs to improve women’s health and well-being.

## 1. Introduction

Urinary incontinence (UI) is a common condition impacting millions of women globally with significant emotional and societal consequences [1]. It is well-defined as the unintentional loss of urine, significantly impairing a person’s quality of life (QoL) and potentially harming their professional lives [2]. The prevalence of UI varies across different Arab countries being highest in Egypt (54.8%) followed by Palestine (43%), Saudi Arabia (41.4) and Qatar (20.7%) [3,4,5,6]. In Jordan, the prevalence of UI is high in rural areas, with rates ranging from 40% to 55.1% among women aged 50–65 [7,8,9].

The type of UI can be stress, urge or combined UI [10]. Identifying the type of UI is crucial for effective management and treatment. The frequency and volume of urine loss are key factors in determining the severity of the condition. Most women with stress incontinence and little volume loss generally deal with their condition without seeking medical help. However, in elderly women, the prevalence of urge UI is considerable, and leakage can evolve from sporadic to regular [9].

Women with stress and urge incontinence are more prone to having a negative QoL [11,12]. Full examination and treatment of UI are impossible without considering the affected woman’s entire QoL. Women’s health-seeking behavior to control urine incontinence is influenced by the severity of the problem and its effect on their QoL [8]. Therefore, preventing UI in women is crucial, especially since effective therapies are limited and the risk increases with age [13].

In the context of Jordan, the socio-economic and cultural landscape is marked by pronounced differences between rural areas and urban centers. These disparities encompass various aspects such as economic opportunities, educational resources, healthcare accessibility, and cultural norms, all of which play a crucial role in shaping health outcomes and lifestyle behaviors [14,15,16]. Rural areas, for instance, often face significant challenges due to limited access to healthcare facilities, lower levels of educational attainment, and cultural practices that differ substantially from those in urban centers. These challenges are exacerbated by infrastructural deficiencies and economic hardships, further contributing to the health disparities observed between these regions. Specifically, in the context of UI, these socio-economic and cultural factors may lead to distinct patterns in prevalence, management, and overall impact on QoL.

Given the high prevalence of UI in rural area of Jordan and the lack of clinical data on its prevalence in Jordan center, the aim of the study is to assess the prevalence of UI in a demonstrative sample of Jordanian women living in the center of Jordan and investigate the correlation between UI and different demographic and gynecological factors, as well as their impact on women’s QoL.

The predictors of UI in low- and middle-income regions have not been extensively studied. Gathering reliable data from various regions with differing social characteristics is essential for developing and implementing effective prevention strategies. As a result, this study’s findings can increase patient awareness of potential risk factors and preventive measures, providing women with knowledge about modifiable risks. These insights may also be applicable to other countries with similar cultural, social, and demographic features.

## 2. Study Design

### 2.1. Targeted Population

A demonstrative size sample of women in the age group above 20 years living in the center of Jordan were approached. The women with UI symptoms who attended any of three different gynecology clinics in central Jordan were approached. A urogynecology physical examination confirmed the diagnosis of UI through the application of a simple and non-invasive Bladder Stress Test and a pelvic exam. The Bladder Stress Test involves asking the patient to cough or perform a Valsalva maneuver while the healthcare provider monitors for any involuntary urine leakage. The pelvic exam assesses the condition of the pelvic floor muscles and checks for signs of pelvic organ prolapse, both of which can contribute to UI.

### 2.2. Ethical Considerations

The women were requested to participate in a self-questionnaire evaluation to quantitively and qualitatively reflect on their experience. The study was conducted in accordance with the guidelines of the Declaration of Helsinki and approved by the Institutional Review board Committee at Amman Arab University, Amman-Jordan. Informed consent was obtained from all women involved in the study (Approval number 0123/08 at 12 August 2022).

Women were asked to rate their experiences anonymously and honestly. The evaluation was completed independently by each woman and all participants had the freedom to withdraw at any point without consequences. The instructions, as well as the study’s goals and structure, were included in the consent form that the women had to read and agree with before participating. Personal information was kept secured and used solely for the current study.

### 2.3. Methodology

A cross-sectional study was performed from September to December of 2022. An online survey hosted on Google Forms or face-to-face interviews were used. The choice depended on the participant’s situation. If a participant was unable to complete the survey online, the survey was conducted through a face-to-face interview instead. The Arabic translated validated short forms of the Urogenital Distress Inventory (UDI-6) and the Incontinence Impact Questionnaire (IIQ-7) were adopted. These were found to be reliable tools for investigating UI and its effect on QoL in Tunisian women [17]. They have been reported to have a high internal consistency (Cronbach’s α > 0.9). The two questionnaires were combined into one form in addition to the demographic and gynecological data. The UDI-6 allows for the diagnosis of UI, while IIQ-7 estimates the severity of UI, and its influence on QoL in women who present with UI. Both questionnaires have six aspects, each on an ordinal scale with possible responses of “never”, “a little”, “moderately”, and “a lot”. A value of zero is assigned for the response “never”, 1 for “a little”, 2 for “moderately”, and 3 for “a lot”. Descriptive statistics for each aspect and the total scores of both questionnaires were calculated. When the values from each of the questions are added together, the total score varies from zero to 18. While each aspect’s raw scores are translated into a score ranging from zero to one hundred. The overall score was categorized as follows: 0–6 mild, 7–12 moderate, and 13–18 severe.

The initial section collected sociodemographic data from the participating women, which included their age, marital status, education level, body weight, height, smoking habits, and monthly income. Body Mass Index (BMI) was then determined using the provided height and weight and Equation (1). BMI serves as a standardized method that offers a more thorough evaluation of body composition and enables consistent monitoring over time. Furthermore, it allows healthcare providers to give more personalized advice on lifestyle changes, such as weight management strategies, to reduce the risk of UI, which may not be evident from weight alone. The BMI classified the participants into underweight, normal range, overweight, and obesity.

(1) $$BMI=\frac{Weight\left(Kg\right)}{Height\left(m\right)2}$$

The second section focused on gynecological history, covering the number of vaginal births, miscarriages, and cesarean sections. The third section addressed the prevalence of UI, examining factors such as urination frequency, urgency, leakage during physical activity, amount of leakage, difficulty in emptying the bladder, and any associated lower back pain or discomfort. The fourth section assessed the impact of UI on QoL, including the ability to perform household chores (such as cooking, laundry, and cleaning), engage in physical activities (like swimming, walking, or praying), participate in recreational activities (such as going to the cinema or a concert), travel by bus or car for over 20 min, and join social events. It also examined how UI affects women’s feelings, potentially leading to increased frustration. Specific research variables related to the questionnaire questions were identified.

### 2.4. Data Processing and Statistical Analysis

The SPSS software version 25 was used for data analysis after exporting responses from Google Forms into Microsoft Excel Sheets (IBM Corporation, Armonk, NY, USA). To establish the significance of each quality feature measured, a two-tailed t-test analysis within subjects was undertaken, with a Cronbach’s alpha value of 0.975 and a significance threshold of *p* < 0.05. To investigate the associations between the demographic data and gynecological history data with UI, multivariate regression analysis was carried out. The study was conducted using IBM SPSS software, version 25. Regression analysis was used to evaluate the independent variables’ respective contributions while adjusting for other variables. 95% confidence intervals (CI) and odds ratios (OR) were computed. At *p* < 0.05, statistical significance was established.

The statistical significance of the distribution of participants across the score grid categories was evaluated using the Chi-Square Goodness-of-Fit test. The analysis was conducted with Python, calculating the Chi-Square statistic and *p*-value. A significance threshold of *p* < 0.05 was applied. The results revealed a Chi-Square statistic of 0.0 and a *p*-value of 1.0, indicating no statistically significant difference in the score distribution.

## 3. Results

### 3.1. Participant’s Demographic

Table 1 shows the sociodemographic and gynecological data of the participated women. A total of 128 women participated in the study. Most of the participants 45.3% (N = 58) were in the age range of 30–40 years, followed by 31.3% (N = 40) participants who were less than 30 years old, 19.5% (N = 25) with the age 40–50 and 3.1% (N = 4) with the age 50–60 years. The vast majority of the participants (96.9%, N = 124) were married, non-smoking, and held bachelor’s degree (58.6%, N = 75). Most of the participants (46.1%, N = 59) were overweight or obese (39.1%, N = 50). Only 25% (N = 26) of the participants had an income salary of more than 1000 Jordanian dinar (JOD), while 35% (N = 46) and 39% (N = 50) had either less than 500 or from 500 to 1000 JOD, respectively.

### 3.2. Participant’s Gynecological History

The majority of the women 74.2% (N = 95) had had at least one vaginal birth. Whereas 24.2, 19.5, and 18.8% of women had had three or two or four vaginal births. While 36.7% (N = 47) of the women had at least one cesarean delivery and 43% (N = 55) had at least one abortion.

### 3.3. UI Prevalence

Table 2 shows an analysis of the items in the questionnaire and the total sum of the responses based on the frequency and the scoring grid for the questions 1–6. It was found that most of the participants (54.33%, N = 69) had mild UI, 35.43% (N = 45) had moderate UI, whereas 10.24% (N = 13) had severe UI. The estimated prevalence of UI was (54%).

### 3.4. Risk Factors Associated with UI

Among the investigated demographic and gynecological aspects, BMI was the sole statistically significant factor linked to UI. The odds ratio (OR) analysis showed that obese patients have the highest risk to develop UI compared to healthy weight patients (OR 35, 95% CI 2.577–475.308, *p* < 0.05). However, the multivariate regression analysis of the factors associated with severe UI showed a significant correlation with smoking and vaginal births. OR was 1.733 (95% CI 0.339–8.860, *p* < 0.003) for smoking and 1.586 (95% CI 0.181–1.895, *p* < 0.02) for vaginal births.

### 3.5. Effect of UI on the QoL

Table 3 shows the total sum of the responses on questions 7–12. It was found that most of the participants; 58.27% (N = 74) had null to mild effect in the QoL, 36.22% (N = 46) of them had moderate effect, whereas 6.30% (N = 8) had severe effect. Complaints were mostly about physical activities that need body movement. Swimming, walking, praying, or shopping were the most affected activities; 42.2% (N = 52) highlighted the negative effects on these activities. In addition, UI is perceived as an embarrassing disease that restricts the social outdoor participation of women where 39.8% (N = 51) felt depressed because of UI, although the influence on QoL appears to be null to mild in general.

Moreover, the unpaired t-test results showed that the QoL is directly proportional to the severity of the UI. The higher is the severity of the UI, the higher its impact on the QoL of the women.

## 4. Discussion

This study’s results offer important insights into the sociodemographic and gynecological traits of women with UI, as well as the risk factors linked to the condition. The study found that UI is a common condition among women, with most of the participants experiencing mild or moderate UI, while only a small proportion had severe UI. This finding highlights the need for healthcare providers to be aware of UI and the importance of early detection and treatment to prevent the condition from worsening. Our study’s methodology prioritized items to assess the prevalence of UI in the center of Jordan, which has not been estimated previously.

The estimated prevalence of UI in central Jordan (54%) was nearly identical to the reported rate in rural northern Jordan seven years ago (55%) [7] and notably higher than the rate reported in rural southern Jordan six years ago (40%) [8]. This trend suggests that the number of UI cases is on the rise, highlighting the need for increased attention and intervention to prevent further escalation.

The study found that the majority of participants were in the age range of 30–40 years, married, non-smoking, and had a bachelor’s degree. Additionally, most of the participants were overweight or obese, and had had at least one vaginal birth. The high prevalence of overweight or obesity in this population is a concerning finding as this is a modifiable risk factor that can be addressed through lifestyle modifications such as diet and exercise [18,19]. BMI was the only statistically significant factor correlated with UI. Obese patients had the highest risk (OR 35, 95% CI 2.577–475.308, *p* < 0.05) compared to those with a healthy weight. This finding is consistent with previous studies that have identified obesity as a risk factor for UI, as excess weight can place additional pressure on the pelvic floor muscles and weaken them, leading to UI [20].

Interestingly, the multivariate regression analysis identified smoking and vaginal deliveries as significantly associated with severe UI. Although the proportion of women who smoke in this study was relatively low, the results demonstrated that smoking remained a significant risk factor for severe UI within our population. This finding aligns with the existing literature and underscores the substantial impact of smoking on the development of severe UI [21]. Previous studies have recognized smoking as a risk factor for severe UI [22,23].

This finding highlights the importance of healthcare providers considering additional factors that may contribute to the severity of UI in addition to its prevalence. Smoking is known to increase the risk of chronic obstructive pulmonary disease (COPD) and other chronic conditions, which can lead to chronic coughing that may weaken the pelvic floor muscles and contribute to the severity of UI [16]. In addition, nicotine, which is the major component of cigarettes, has neuropharmacological actions that initiates large spontaneous bladder contractions [24].

The effects of vaginal childbirth and cesarean delivery are still debated, and neither appeared to have a significant correlation with UI. However, vaginal childbirth showed clear significant links with the severity of illness. Vaginal deliveries led to perineal damage that aggravated the UI [25]. Moreover, vaginal births can cause damage to the pelvic floor muscles, leading to UI [26].

Additionally, involuntary urine loss can be both hygienically and socially distressing for patients. The study revealed that UI significantly affects women’s QoL, particularly hindering physical activities involving body movement. Activities such as swimming, walking, praying, and shopping were notably impacted by UI. This finding is consistent with previous research, which has demonstrated that UI impairs women’s ability to engage in daily activities, potentially leading to social isolation and decreased involvement in recreational activities [27,28]. Furthermore, despite the impact of UI on QoL being relatively mild, women sought clinical treatment due to religious practices. As Muslims, they need to pray five times a day and must be dry before and during each prayer. Even a single instance of urinary leakage per day can be a significant source of discomfort for these women. This issue has been noted in several UI studies conducted in Arab countries, including Qatar [6], the United Arab Emirates [29], and Egypt [4].

Despite the negative impact of UI on daily activities, the influence on QoL appears to be mild in general, which is consistent with previous studies [30,31]. It is possible that women with UI have adapted to their condition and have learned to manage their symptoms, thus reducing the impact on their QoL.

Furthermore, the results of the unpaired t-test showed that the severity of UI is directly proportional to its impact on the QoL of women. This finding aligns with previous research, which indicates that women with severe UI experience a lower QoL compared to those with mild to moderate incontinence [32,33]. It highlights the importance of early interventions and management of UI to prevent its progression and reduce its negative impact on women’s QoL.

In conclusion, UI is a widespread and challenging problem for many women, particularly among those who are obese, smokers, or have experienced vaginal births. The significant physical and emotional toll that UI imposes underscores the importance of healthcare providers being vigilant about the associated risk factors. By actively addressing modifiable factors such as weight management, smoking cessation, and postnatal care, healthcare professionals can play a pivotal role in early intervention and management of UI, ultimately improving the quality of life for affected women.

Moreover, the development of tailored care plans that include patient education, behavioral therapies, and access to appropriate medical treatments is essential. Such comprehensive strategies not only alleviate the immediate symptoms but also contribute to long-term health benefits. To further strengthen these efforts, prospective cohort studies are essential to evaluate the effectiveness of various interventions and to understand their impact on reducing the negative consequences of UI on women’s physical, emotional, and social well-being. These studies will provide valuable insights that can inform public health policies and enhance the support systems available to women struggling with UI.

Furthermore, it is vital to accurately identify and target the most severely affected patients for research and treatment to allocate resources effectively. However, because UI is a stigmatized condition in many societies, gathering consistent epidemiological data can be challenging. Therefore, future research with larger sample sizes is necessary to produce more accurate estimates of UI prevalence and its correlations, as well as to deepen our understanding of the risk factors and underlying causes of the condition. Ultimately, women in central Jordan represent a key demographic for prevention and health awareness initiatives.

## 5. Conclusions

This study highlights a significant public health concern, with more than half of the participants experiencing UI symptoms. These symptoms not only detract from their QoL but also create physical discomfort during activities such as exercise and daily chores, leading many to seek medical or therapeutic intervention. The study identified key factors associated with the prevalence of UI, including obesity, smoking, and a history of vaginal delivery, all of which underscore the multifactorial nature of this condition.

Given these findings, there is a clear and urgent need for targeted interventions within women’s health centers in central Jordan. These centers should prioritize the development and implementation of comprehensive programs focused on both the prevention and management of UI. This could include educational campaigns, support groups, and individualized treatment plans aimed at mitigating risk factors like obesity and smoking, as well as providing specialized care for women who have experienced vaginal deliveries. By addressing these needs, health centers can significantly improve the quality of life for women affected by UI, fostering a healthier and more informed community.

## Figures and Tables

**Table 1:** The sociodemographic and gynecological data of the participated women.

The Variables	Frequency N	Percentage (%)
**Part 1: The Sociodemographic Data**		
Age		
20–30	40	31.3
30–40	58	45.3
40–50	25	19.5
50–60	4	3.1
Above 60	1	0.8
Marital status		
Married	124	96.9
Single	4	3.1
Education level		
Master’s degree	4	3.1
Bachelor of Science	75	58.6
Diploma	30	23.4
High school	18	14.1
Other	1	0.8
Body mass index		
Obese (30 and above)	50	39.1
Overweight (25.0–29.9)	59	56.1
Healthy (18.5–24.5)	18	14.1
Underweight (below 18.5)	1	0.8
Smoking		
No	116	90.6
Yes	12	9.4
Income salary		
<500 JOD	46	35.9
>1000 JOD	32	25.0
500–1000	50	39.1
**Part 2: The gynecologically data**		
Number of vaginal births		
0	33	25.8
1	9	7.0
2	25	19.5
3	31	24.2
4	24	18.8
5	6	4.7
Number of Cesarean deliveries		
0	81	63.3
1	29	22.7
2	10	7.8
3	5	3.9
4	0	0
5	3	2.3
Numbers of miscarriages		
0	73	57.0
1	26	20.3
2	21	16.4
3	7	5.5
4	1	0.8

**Table 2:** Item analysis of each response for questions 1–6 with the percentage of the total sum of the responses and the frequency based on the score grid used to classify the severity of UI.

The Variables	Percentage of Responses (Frequency)			
Part 3: Prevalence of UI	Never	Little	Medium	A Lot
1. Do you have increase in urination frequency?	14.8% (19)	33.6% (43)	34.4% (44)	17.2% (22)
2. Do you feel the urge to urinate?	19.5% (25)	35.2% (45)	33.6% (43)	10.9% (14)
3. Do you have incontinence during high physical effort (such as coughing, sneezing, movement)?	19.5% (25)	38.3% (49)	31.3% (40)	10.9% (14)
4. Do you have mild incontinence (few drops of urine)?	24.2% (31)	36.7% (47)	29.7% (38)	9.4% (12)
5. Do you have urine sack (difficulty in emptying the bladder)?	29.7% (38)	33.6% (43)	28.1% (36)	8.6% (11)
6. Do you have lower back pain/ bottom vulva pain?	14.1% (18)	41.4% (53)	31.3% (40)	11.7% (15)
Score Grid	Total frequency (N)		Percentage (%)	
0–6 (mild UI)	69		54.33	
7–12 (moderate UI)	45		35.43	
13–18 (severe UI)	13		10.24	

**Table 3:** Item analysis of each response for questions 7–12 with the percentage of the total sum of the responses and the frequency based on score grid used to determine the effect of UI in the QoL.

The Variables	Percentage of Responses (Frequency)			
Part 4: Impact of UI on QoL	Never	Little	Medium	A Lot
7. Does the incontinence affect your ability to do chores (cooking, house cleaning, washing cloths)?	23.4% (30)	33.6% (43)	33.6% (43)	9.4% (12)
8. Does the incontinence affect your physical activities that need body movement like swimming or walking or praying or shopping?	22.7% (29)	42.2% (54)	28.1% (36)	7% (9)
9. Does the incontinence affect the sedentary recreation activities?	28.1% (36)	38.3% (49)	27.3% (35)	6.3% (8)
10. Does the incontinence affect your ability to ride a car or bus for more than 20 min?	20.3% (26)	38.3% (49)	31.3% (40)	10.2% (13)
11. Does your incontinence affect your social outdoor participation?	24.2% (31)	35.2% (45)	34.4% (44)	6.3% (8)
12. Do you feel depresses due to incontinence?	25% (32)	39.8% (51)	28.9% (37)	6.3% (8)
Score Grid	Total frequency (N)		Percentage (%)	
0–6 (null-mild effect)	74		58.27	
7–12 (moderate effect)	46		36.22	
13–18 (severe effect)	8		6.30	

## Data Availability

The raw data supporting the conclusions of this article will be made available by the authors on request.

## Abbreviations

UI: urinary incontinence; QoL: quality of life; UI: IIQ-7: Incontinence Impact Questionnaire short form; UDI-6: Urogenital Distress Inventory short form.
